# Therapeutical Notes

**Published:** 1899-12-30

**Authors:** 


### THERAPEUTICAL NOTES.

### Recurrent Epistaxis.

Rendu recommends the following :—

℞. Antipyrin, gr. vii.

Tannin, gr. xv.

Sacchari albi, ʒijss.

M. S. To be used locally.

—*New York Med. Jour.*, Feb. 25.

### Orexin in the Vomiting of Pregnancy.

F. Hermanni found in nine cases remarkable results from the use of orexin tannate, which is tasteless, and can be given in pill or powder. Even in the worst cases it will often check vomiting. It should be followed by a small draught of water when this is possible.

℞. Orexin tannate gr. iv.
fiat pilula bis die sum.

### An Expectorant.

℞. Apomorphinæ hydrochloratis, gr. jss.

Acidi Hydrochlorici, mxxv.

Syrupi aromatici, ℥jss.

Aq. destillatæ, ℥vi.

M. S., ʒss. quartis horis.

—*Merck's Archives*, February.

